# Biomaterials enhancing performance of cell and nucleic-acid therapies: An opportunity in the brain

**DOI:** 10.1016/j.bbiosy.2021.100036

**Published:** 2021-12-29

**Authors:** Christopher Lally, Kieran Joyce, Abhay Pandit

**Affiliations:** a CÚRAM SFI Research Centre for Medical Devices, National University of Ireland Galway, Galway, Ireland; b School of Medicine, National University of Ireland Galway, Galway, Ireland

**Keywords:** Cell therapy, Gene therapy, Brain, ATMP

## Abstract

The brain has limited innate ability to promote repair, regeneration and functional recovery following injury, disease, or developmental disorder. Although cell and gene therapies have significant potential in the brain, no single treatment is likely to succeed in isolation. Here we discuss the current state of the art in cell and nucleic-acid-based neurotherapeutics and argue for the development of combination therapies that use biomaterials to help overcome the current limitations of cell and gene therapies alone.

## Highlights

• No effective treatment for neurodegenerative disease.
• Biomaterials are a new hope for cell and gene therapies.
• Biomaterial-based combination treatments are the future of cell and gene therapies.

## Introduction

For many of the most chronic and debilitating neurological disorders, such as brain injury, neurodegenerative disease and congenital disorders of the brain, no robust preventative measure or effective disease-modifying therapy is currently available. With an ageing global population, these unmet needs will only become more pressing.

Amongst the most promising new therapeutic candidates are cell and gene therapies. To date, 18 cell or gene therapies have been approved by the European Medicines Agency (EMA), under the designation of “Advanced therapy medicinal products” (ATMPs). 11 have a current marketing authorisation at the time of writing: 7 gene therapies and 4 cell therapies. Only 2 of these target the central nervous system (CNS) – the gene therapies Zolgensma®, for spinal muscular atrophy, and Skysona®, which targets an inborn error of metabolism that results in cerebral adrenoleukodystrophy. A recent study estimated that only 11 new neuro/neuromuscular cell and gene therapies were currently being investigated in clinical trials [1].

## Combination therapies

The EMA defines Combination ATMPs as therapies that use a cell or gene therapy to achieve their effects alongside a component that does not rely on pharmacological, immunological, or metabolic means to exert its effect – This includes devices such as biomaterial cell scaffolds or nanoparticles for gene therapy delivery.

The definition of “biomaterial” in the context of Combination ATMPs is broad-ranging, encompassing both natural and synthetic materials in various forms such as gels, particles, and more structurally complex constructs that aim to restore aspects of normal tissue architecture from subcellular to gross anatomical scale. Many biomaterials have inherent therapeutic utility in addition to their ability to support cell and gene therapies, as is discussed further below. However, the European Union (EU) clinical trials register currently lists no combination ATMP trials for neurological applications. Combination ATMPs for the brain, therefore, represent an underutilised approach. The utility of these systems will depend on the design of appropriate biomaterials for specific uses within the brain.

## Biomaterials for the brain

Biomaterials for the brain must meet several general requirements. Ideally, the material should match the mechanical properties of the administration site, as softer materials tend to be less stable and stiffer materials may result in gliosis [2]. Degradation rate must also be controlled – rapid degradation may impair functionality, but non-degradable materials are associated with chronic inflammation, scarring and neuronal loss [2]. The material should not be cytotoxic, immunogenic or cause undue secondary injury to nearby cells due to swelling [2]. A wide range of natural and synthetic biomaterials have been used in the brain in the form of hydrogels, particles and electrospun fibres. The specific material selected is highly dependant on the intended site of administration and desired use. Hydrogels and particles can be delivered via injection to the brain parenchyma, which results in less trauma than other forms of surgical implantation, but offer less control over the resulting structure than preformed biomaterials [2].

Restoring structure and function in the brain will require fine control over tissue architecture from subcellular to gross anatomical scale. Parkinson's disease is an instructive example – In Parkinson's disease, dysfunction and loss of dopaminergic neurons connecting the substantia nigra and striatum results in multi-centimetre scale lesions. Slowing disease progression and restoring function would require promotion of endogenous axonal growth and synaptogenesis or engraftment of cells capable of functional integration. Biomaterials such as growth-factor laden collagen scaffolds can help “bridge” these gaps by providing biochemical and physical guidance cues over relevant distances [3].

Biomaterials can provide structural cues essential for restoring brain architecture. Recent advances in inkjet, micro-extrusion and laser-assisted bioprinting have shown that fine control over biomaterial structure is achievable while maintaining strong cell survival and tuneable mechanical properties. Additionally, electrospinning of fibres allows for anisotropic, patterned materials to be generated that provide topographical cues for directed cell growth, such as axon or neurite guidance [4].

Many natural biomaterials have intrinsic bioactive effects in and of themselves – For example, fibrin, collagen, laminin, and hyaluronic acid bear versatile binding sites that allow them to interact with both grafted cells and host brain tissue and provide chemotactic cues for the migration, invasion, and retention of graft/host cells [5]. In effect, biomaterials allow for the design of a favourable microenvironment at their site of administration within the brain.

## Cell therapies

**Fig. 1.**
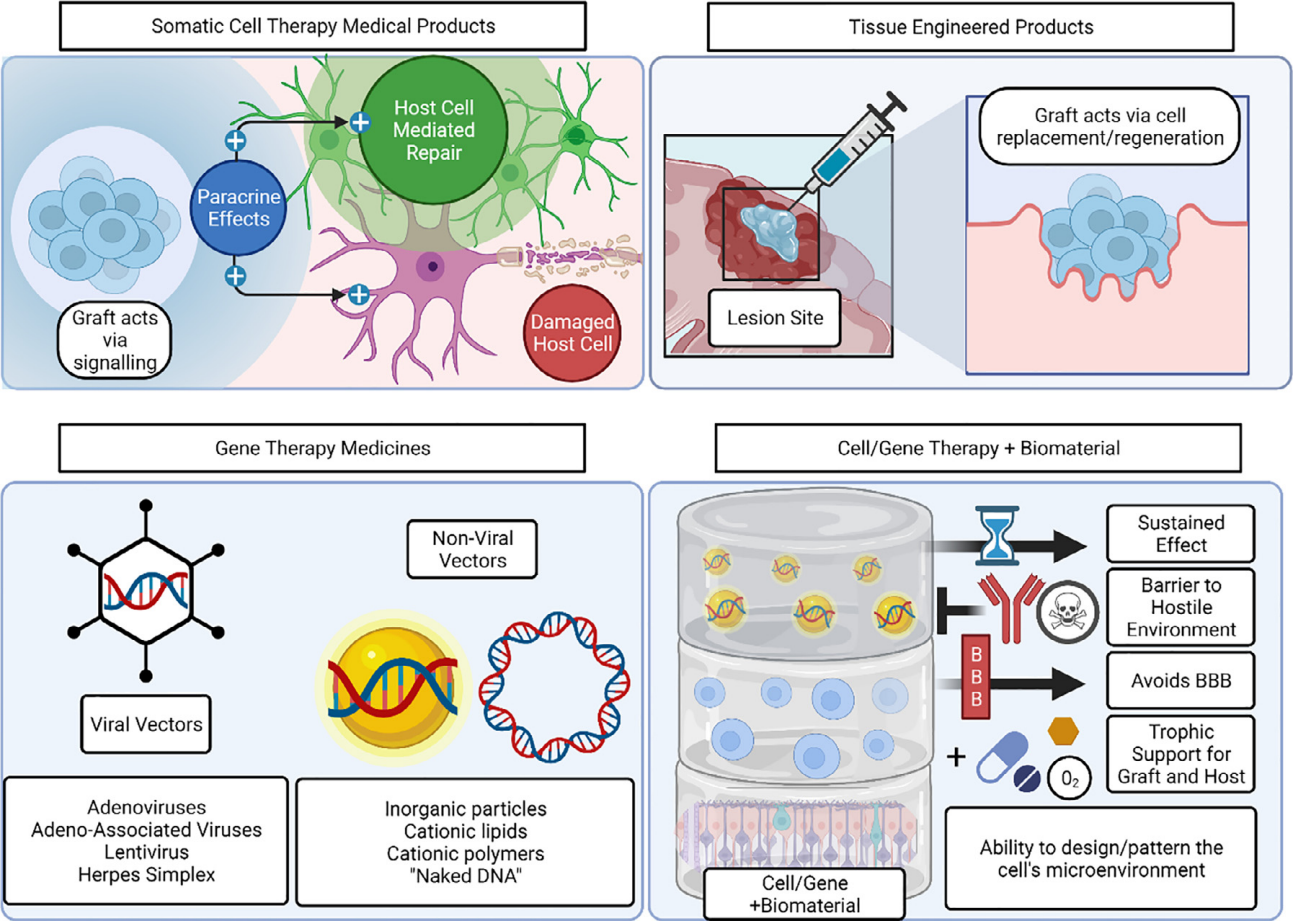
Cell and nucleic-acid therapies can benefit from the use of biomaterials as a supporting technology. Biomaterials can increase the dose of cell or nucleic-acid retained at the site of action, mitigate cell survival or nucleic-acid degradation challenges, and help co-administer other therapeutics to enhance cell and nucleic-acid therapy. .

Cell therapies are divided by the EMA into somatic-cell therapy medical products (sCTMPs) and tissue-engineered products (TEPs). In sCTMPs, engrafted cells are not intended to replace/regenerate lost tissue directly. Instead, they act through the pharmacological, immunological, or metabolic action of their cells or tissues. Tissue-engineered products use engrafted cells or tissues to regenerate, repair or replace damaged host tissue (Fig. 1).

The adult brain has only a small resident population of stem cells, which show limited ability to proliferate or migrate within the brain to regenerate cells and tissues lost to injury or disease [6]. Cell therapies may aim to replace lost cells directly, but more commonly aim to provide trophic support to resident cells, mitigate neuroinflammation or secrete a therapeutic agent [6].

Cell therapies face several key challenges. As for any neurotherapeutic, delivery to the brain is complicated by significant losses due to the blood-brain barrier. Even when locally administered, the dysfunctional brain is a hostile environment for transplanted cells due to the presence of toxic metabolites, inflammation and disruption of normal tissue architecture in the form of a glial scar or cyst. Where restoration of function depends on the integration of grafted cells into host neuronal networks, cells may need substantial pre-treatment, genetic modification, or pharmacological manipulation to promote axon outgrowth and synaptogenesis in this environment [6].

For example, following stroke, a large fluid-filled cavity remains, bounded by a glial scar and dysfunctional (but potentially salvageable) cells. Chronic inflammation and catastrophic loss of tissue architecture (from subcellular features such as cell adhesion sites to anatomical scale features such as vasculature) make this a uniquely challenging site for cellular regeneration [2]. However, injectable hydrogels such as collagen, fibrin, Matrigel® and polyethylene glycol can provide a rationally designed microenvironment that can support the survival, attachment, proliferation and migration of engrafted cells and surrounding host tissues within the brain [5]. Many hydrogel biomaterials allow for functionalisation with drugs and biomolecules that can offset oxygen or nutrient deprivation, provide trophic cues, or release therapeutics to the host tissue [5]. Thus, cell/biomaterial combination therapies have significant potential to address complex, multifactorial therapeutic needs in the brain.

## Nucleic-acid therapies

Recombinant nucleic acids are used to regulate, repair, add or delete a gene sequence in this approach. Nucleic-acid therapies are a promising therapeutic option in cases with a robust inheritable component, such as Huntington's disease, Amyotrophic lateral sclerosis (ALS) or Parkinson's disease.

Nucleic-acid therapies aimed at correcting loss-of-function gene defects primarily rely on adeno-associated viruses (AAVs) delivered to the brain intracerebrally or intrathecally [7]. Promising results were first obtained in the mid-to-late-2000s using AAVs to target the defective ASPA gene (responsible for Canavan disease), Parkinson's disease, the PPT1 gene (responsible for late infantile neuronal ceroid lipofuscinosis), and spinal muscular atrophy [8]. Retroviruses, lentiviruses, herpes simplex and adeno-associated viruses have also been used [8]. Lentiviruses can infect both proliferating and quiescent cells, including neurons, where they are incorporated into the host DNA to provide stable and sustained gene expression. They have been tested in preclinical models of Alzheimer's disease and Parkinson's disease. Adenoviral vectors are useful where integration into the host genome is undesirable and allow for transient expression of larger genes (35–40 kb). However, they are limited by their immunogenicity and cytotoxic potential [8]. They have mainly been investigated in tumour therapy and animal models of Parkinson's disease and Huntington's disease. Herpes simplex vectors are inherently neurotropic, migrate from peripheral injection sites via axons to take up residence in the CNS, and have a large (∼150 kb) carrying capacity that allows multiple transgenes to be delivered. They are limited by the rapid silencing of their gene expression, and their potential cytotoxicity must be carefully managed, although this can be useful in neuro-oncology applications [9].

Nonviral vectors avoid or lessen many of the risks associated with viral vectors, typically at the cost of lower transfection efficiency. Nonviral gene delivery approaches are inherently reliant on biomaterials, such as nucleic-acid carriers, to mitigate against degradation of the payload during delivery and promote uptake into target cells. These include inorganic nanoparticles such as gold, calcium phosphate or silica, cationic lipid nanoparticles, emulsions, peptide-based carriers, and polymers [9].

For either approach, viral or nonviral, the main challenges of gene delivery to the brain are the rapid degradation of the genetic payload, insufficient gene dose at the site of administration, off-target gene expression and transient expression of therapeutic genes [10]. Biomaterials can enhance the effectiveness of gene therapies by providing a barrier between the genetic payload and host-mediated degradation, helping to retain the vector at the site of action, and providing a sustained release for prolonged-expression of transiently-expressed genes [10]. This could make biomaterial-mediated gene delivery more efficient while also limiting the need for immunosuppression or repeat administration to maintain effectiveness, lessening the risk of opportunistic infection or iatrogenic injury to the brain [10].

## Conclusion

Biomaterials have strong potential as an enabling technology for cell and gene therapies. We propose that the current lack of combined ATMP approaches in clinical trials represents a significant opportunity for biomaterials researchers to contribute to the development of effective neurological therapies. The development of new medical devices incorporating cell, gene and biomaterial approaches may help address the complexity of neurological disease, and we anticipate increased use of multimodal approaches in clinical trials over the coming years.

## Declaration of Competing Interest

The authors declare that they have no known competing financial interests or personal relationships that could have appeared to influence the work reported in this paper.
